# A Mixed-Methods Study of Policymakers’ Adoption of AI to Support Use of Research Evidence: Implications for Artificial Intelligence in Prevention Policy

**DOI:** 10.1007/s11121-026-01925-z

**Published:** 2026-05-02

**Authors:** D.Max Crowley, Jonathan Wright, Alex Winters, Damon Jones, Jessica Pugel, Patrick O’Neill, Bethany Shaw, Sarah Hamel, Elizabeth Long, Michael Donovan, Taylor Scott

**Affiliations:** Edna Bennett Pierce Prevention Research Center, The Pennsylvania State University, University Park, PA USA

**Keywords:** Artificial intelligence, Public policy, Clearinghouses

## Abstract

Policymakers are increasingly adopting artificial intelligence (AI) tools to support legislative decision-making, yet there is limited empirical understanding of how these technologies are used and the implications for evidence-based policymaking. General-purpose AI tools, such as large language models (LLMs), present both opportunities for improved efficiency and risks related to misinformation and lack of transparency. This study examines state legislators’ use of AI in policymaking and introduces the AIRE Protocol (AI for Informed and Responsible Evidence-use), a structured framework for developing specialized AI tools grounded in validated evidence. We demonstrate the application of the AIRE Protocol through the development of the Results First AI Assistant, designed to enhance policymakers’ access to the Results First Clearinghouse. A mixed-methods approach was used. Forty-five US state legislators participated in live interviews to assess AI adoption patterns, perceived benefits, and concerns. The AIRE Protocol guided the rapid prototyping and iterative development of the AI assistant, with input from policymakers, national policy organizations, and technical experts, resulting in tailored evidence based recommendations. While policymakers expressed interest in AI tools for improving access to information under time constraints, they also raised concerns regarding transparency, reliability, and appropriate use. Our findings suggest that AI tools tailored to policymakers’ needs—developed using frameworks like AIRE—will facilitate the integration of validated evidence into legislative decision-making while addressing ethical and practical concerns associated with generalized AI solutions.

Policymakers are increasingly incorporating artificial intelligence (AI) tools into their decision-making processes, with commercial large language models (LLMs) like ChatGPT gaining traction for their ability to quickly synthesize vast amounts of information (Babšek et al., 2025). While the adoption of AI in policymaking is often celebrated for enhancing efficiency and improving access to information, it raises important concerns about the integrity, transparency, and reliability of the information that underpins legislative decisions (Neumann et al., 2024). The integration of generalized AI tools, trained on expansive but uncurated datasets, introduces significant risks—particularly the potential for disseminating misinformation, reinforcing bias, and offering recommendations misaligned with scientific evidence (Guedes & Júnior, 2024). These risks are especially concerning in prevention policy, where decisions about interventions targeting children, families, and communities require a foundation of rigorous, validated research (Best et al., 2024). The integration of AI into policymaking is part of a broader trend of digital transformation in the public sector (Osborne et al., 2022; Ruvalcaba-Gomez, 2023). Over the past two decades, governments worldwide have increasingly leveraged data analytics, machine learning, and AI to improve service delivery, enhance transparency, and streamline decision-making (Dziundziuk et al., 2024). From predictive policing algorithms to AI-driven welfare assessments in Europe and Asia, the applications are diverse, albeit often accompanied by debates about fairness, privacy, and accountability (Li et al., 2023; van Noordt et al., 2023). The USA has similarly invested in AI for national security, healthcare policy analysis, and, now, legislative processes. Understanding this global and historical trajectory is essential to contextualize both the opportunities and challenges AI presents in prevention policy (Brundage et al., 2024; Hunt et al., 2020; Johansson, 2025). The deployment of AI in government settings does not occur in a regulatory vacuum. In the United States, recent federal directives, including Executive Order 14,179 (Removing Barriers to American Leadership in Artificial Intelligence, 2025), reflect a push to advance AI innovation while navigating complex regulatory landscapes. Internationally, the European Union’s AI Act (Future of Life Institute, n.d.) and similar global frameworks aim to standardize ethical AI deployment, emphasizing transparency, data protection, and human oversight. These evolving policies underscore the importance of developing AI tools for policymakers that meet technological standards and align with emerging legal and ethical requirements. Recent developments in government-led AI initiatives underscore both the promise and complexity of integrating AI into public policy and decision-making processes (Mergel et al., 2024). In the United States, the growing emphasis on AI is evident in major federal initiatives, including President Trump’s Executive Order 14,179, Removing Barriers to American Leadership in Artificial Intelligence, which aims to accelerate AI development by eliminating regulatory hurdles while maintaining US technological dominance. Similarly, recent efforts to develop “GSAi,” an AI chatbot designed to streamline federal procurement processes, reflect a broader movement toward using AI to improve government operations. While these initiatives highlight AI’s potential to enhance efficiency and information management in policy contexts, they also raise significant ethical and governance concerns. Critics warn that rapid AI adoption without adequate oversight could undermine transparency, centralize decision-making authority, and erode public trust in democratic institutions (Fan, 2024). These tensions illustrate the critical need for frameworks that balance technological innovation with ethical considerations, ensuring AI systems deployed in policymaking contexts are transparent, accountable, and aligned with public values (Chandra & Feng, 2025). This context reinforces the importance of developing specialized AI tools that are not only efficient but also grounded in validated evidence and designed to support informed, responsible policymaking. The promise of AI lies in its ability to support the policymaking process by delivering timely information and facilitating data-driven decisions. While there has been demonstrated improvement in some AI tools (E.g., Policybot; Milmo, 2025), without mechanisms to ensure the accuracy and relevance of AI-generated outputs, policymakers may unknowingly base critical decisions on flawed information. This issue is not merely theoretical—there is growing evidence that AI tools, if not carefully designed and implemented, can perpetuate existing inequities and amplify misinformation, ultimately undermining efforts to implement effective prevention strategies. Considering these challenges, there is an urgent need to develop AI tools that are not only efficient but also trustworthy, transparent, and grounded in validated evidence. Such tools can play a pivotal role in bridging the gap between research and policy, ensuring that decisions affecting population health are informed by the best available science

## Challenges in Prevention Policy

Effective prevention policy relies on timely access to evidence-based information (Crowley & Scott, 2017; Crowley et al., 2022; Scott et al., 2024), yet policymakers often face significant barriers to obtaining and interpreting the data needed to make informed decisions (Crowley et al., 2020; Scott et al., 2022). Research evidence is frequently dense, technical, and difficult to translate into actionable policy guidance. Moreover, the policy environment is characterized by competing priorities, time constraints, and the influence of political and public pressures, all of which can limit the capacity of legislators to engage deeply with scientific research (Long et al., 2021, 2022, 2024). Existing resources, while valuable, may not be designed with the specific needs of policymakers in mind, resulting in underutilization of evidence-based clearinghouses and other information repositories (Buckley et al.,; 2025; Crowley et al., 2019, 2024; Scott et al., 2023a, 2023b). Focusing on prevention policy offers a critical lens through which to explore the integration of AI into legislative decision-making. Prevention policies—ranging from substance misuse programs to child welfare interventions—often involve complex cost–benefit considerations and have long-term impacts on population health. Given the urgency and complexity of these decisions, timely access to evidence-based information is vital (Scott et al., 2023a, 2023b). Yet, research shows that policymakers frequently lack the tools to efficiently interpret and apply scientific evidence in these contexts (Crowley et al., 2018, 2021a, 2021b; Scott et al., 2022). By addressing these gaps, AI tools designed for prevention policy can improve resource allocation, enhance program effectiveness, and ultimately yield significant public health benefits.

Commercial AI tools offer a potential solution to these barriers by providing quick summaries of complex information. However, these tools are typically general-purpose, lacking the capacity to discern between high-quality, peer-reviewed research and less credible sources (Corti et al., 2023). Without safeguards to ensure the use of validated evidence, policymakers risk relying on information that, while readily accessible, may be incomplete or misleading. In the context of prevention policy—where decisions can have profound and lasting impacts on public health—this reliance on generalized AI tools presents significant risks including ineffective solutions and wasted resources. There is a clear need for specialized AI solutions tailored to the unique demands of the policymaking process, emphasizing both accessibility and scientific rigor.

## Current Study

This study addresses two primary objectives. First, we seek to understand how US state legislators are currently using AI in their lawmaking processes. Through a national survey, we examine the prevalence of AI adoption, the types of tools being used, and policymakers’ perceptions of the benefits and risks associated with these technologies. By identifying the facilitators and barriers to AI use, we aim to provide a comprehensive overview of how technology is shaping contemporary legislative work.

Second, we introduce and apply the AIRE Protocol—AI for Informed and Responsible Evidence-use—as a structured framework for rapidly developing AI tools that prioritize the use of validated scientific evidence. The AIRE Protocol is demonstrated through the development of an AI assistant designed to enhance access to the Results First Clearinghouse (*Results First: Clearinghouse Database*, 2025), an evidence-based program database developed by The Pew Charitable Trusts. By leveraging this resource, the AI assistant aims to provide policymakers with accurate, timely, and actionable information to inform prevention policy decisions.

### Introducing the AIRE Protocol

The AIRE (AI for Informed and Responsible Evidence-use) Protocol was developed in response to the growing need for AI solutions that are both user-centered and evidence-based. AIRE is built on four core principles: informed use, responsible development, user-centered design, and rapid iteration. Informed use emphasizes that AI outputs must be grounded in validated scientific evidence, ensuring policymakers receive accurate and trustworthy information. Responsible development calls for transparency, ethical considerations, and attention to potential biases in AI systems. User-centered design ensures that tools are developed with direct input from policymakers, reflecting their workflows, preferences, and informational needs. Rapid iteration enables timely development and refinement of AI tools, ensuring they remain relevant in fast-paced policy environments.

To illustrate the AIRE Protocol’s application, this study presents a case in which AIRE guides the development of an AI assistant that interfaces with the Results First Clearinghouse. The assistant is designed to streamline access to evidence on the effectiveness and cost–benefit profiles of prevention programs, addressing a key challenge policymakers face in evaluating intervention options. By following the AIRE framework, the development process centers on creating a practical, accessible tool that supports evidence-informed decision-making without sacrificing the speed and convenience that have driven AI’s popularity in policymaking contexts.

### Framework for Prototyping Evidence-Based AI Tools

The AIRE Protocol was developed to address the need for a structured, replicable process for rapidly prototyping AI tools tailored to the unique needs of policymakers. While commercial AI tools offer general knowledge retrieval, they often lack the specificity and reliability required for evidence-based decision-making in prevention policy. The AIRE Protocol seeks to bridge this gap by ensuring that AI tool development is grounded in validated scientific evidence, informed by end-user needs, and sensitive to ethical considerations. Rooted in principles of user-centered design and rapid iteration, AIRE emphasizes the importance of co-creation with policymakers, transparency in algorithmic processes, and the responsible use of data. The protocol provides a practical roadmap for developing AI applications that not only deliver timely information but also enhance the trust and usability necessary for adoption in legislative contexts. This protocol complements existing AI frameworks as well as efforts to optimize interventions (e.g., MOST; Collins et al., 2007). To demonstrate the application of the AIRE Protocol, we applied its framework to develop an AI assistant designed to interface with the Results First Clearinghouse, enabling policymakers access to evidence-based information on prevention program effectiveness.

### Core Pillars of AIRE

The AIRE Protocol is structured around four core pillars that guide the development of evidence-based AI tools for policymakers:

1. Informed use: AI outputs are derived exclusively from validated scientific evidence, ensuring that policymakers receive accurate and credible information. For this project, the Results First Clearinghouse served as the primary data source, offering rigorously evaluated information on program effectiveness.
2. Responsible development: Ethical considerations are prioritized throughout the development process. This includes transparency in how recommendations are generated, efforts to mitigate algorithmic bias, and safeguards to protect user privacy and data security.
3. User-centered design: Policymakers are engaged as co-creators to ensure that the AI tools reflect their informational needs, decision-making processes, and contextual realities. Regular feedback loops are integrated into development to refine usability and functionality.
4. Rapid iteration: Development cycles are designed to be swift and adaptive, allowing for timely deployment of functional prototypes. Iterative testing ensures that tools remain responsive to user needs and evolving policy contexts.

Together, these pillars provide a comprehensive framework for developing AI tools that are practical, ethical, and effective in supporting evidence-based policymaking.

By considering empirical insights from a national survey of state legislators with the development and application of the AIRE Protocol, this study offers a dual contribution to the fields of prevention science and public sector AI deployment. It not only sheds light on current policymaker attitudes and adoption patterns but also provides a replicable framework for rapidly prototyping AI tools that prioritize transparency, ethical use, and evidence-based decision-making. As governments increasingly turn to AI to navigate complex policy landscapes, this research underscores the importance of developing solutions that are both technologically robust and responsive to the unique demands of the policymaking process.

## Methods

The study employed a mixed-methods design, combining qualitative and quantitative data collected through live interviews with state legislators and the application of a structured prototyping process to develop the Results First AI Assistant. The methods are presented in three parts: (1) the design and administration of a national survey with 45 state legislators to explore current AI use, perceived benefits, barriers, and attitudes; (2) a detailed description of the AIRE Protocol framework guiding the development of the AI tool; and (3) a case study illustrating the application of the protocol to create an AI assistant interfacing with the Results First Clearinghouse. By integrating survey data with an applied development process, this methods section provides a comprehensive overview of how user-centered design and evidence-based practices informed both the research and the tool’s creation.

### Legislator AI Adoption Survey

#### Study Design and Participants

This study employed a mixed-methods approach, with the primary data collection consisting of live interviews conducted with state legislators across the United States. A total of 45 legislators participated in the survey component, providing qualitative and quantitative insights into their experiences and perceptions regarding the use of artificial intelligence (AI) in the policymaking process. The sample was intentionally diverse, representing a broad range of geographic regions, political affiliations, years of legislative experience, and committee assignments to ensure a comprehensive understanding of AI adoption across varying contexts.

Participants were recruited through direct contact with legislative offices in 30 US states. Efforts were made to include legislators with varying levels of familiarity and experience with AI to capture a full spectrum of perspectives. Legislators were initially contacted via personalized email invitations describing the purpose of the study and requesting participation. Follow-up outreach was conducted through in-person or phone contacts with legislative staff to facilitate scheduling and confirm interest, resulting in a final response rate of 68%.

#### Survey Instrument: Survey Instrument Description

The survey instrument was adapted from previously validated studies that examined legislative information-seeking and use of research evidence in policymaking contexts (Crowley et al., 2021; Long et al., 2022). These foundational instruments were originally developed in collaboration with state legislators, legislative staff, and intermediaries, ensuring strong relevance to policymaking settings. For the current study, items were revised to reflect emerging trends in artificial intelligence (AI) use in governance while retaining core constructs related to evidence engagement. The survey followed a structured interview format in which participants were guided through both closed-ended and open-ended items by trained interviewers. The survey instrument was developed through an iterative process involving consultation with policy experts, AI developers, and prevention science researchers to ensure content validity and relevance. The primary construct for this study was around current use of AI in the context of larger information gathering and legislative research activities. The survey was pilot tested with a small group of legislators to refine question wording and improve clarity before full deployment.

#### Data Collection and Analysis

Data collection occurred between August 2024 and July 2025 with each interview lasting approximately 15–30 min. Interviews were conducted via video platforms to accommodate legislators from various states and to ensure flexibility given their demanding schedules. With participant consent, all interviews were audio-recorded and professionally transcribed to ensure accuracy and facilitate analysis. The analysis employed a convergent mixed-methods design, integrating both quantitative and qualitative data to provide a better understanding of AI adoption among state legislators. Quantitative data from closed-ended questions were analyzed using descriptive statistics to identify trends in AI use, perceived benefits and risks, and barriers to adoption. The survey protocol was reviewed and approved by the Institutional Review Board of The Pennsylvania State University (IRB Protocol #00021641). All engagement activities complied with legal and ethical guidelines prohibiting lobbying or advocacy.

### Stages of the AIRE Protocol

To guide the development of the Results First AI Assistant, we applied the AIRE Protocol (Artificial Intelligence for Research Evidence), a structured, human-in-the-loop framework designed to ensure that AI tools for evidence translation are grounded in validated data sources, policy-relevant design, and iterative refinement. The AIRE Protocol was implemented in five sequential stages—needs assessment, co-design, prototyping, testing, and deployment—each tailored to the goal of building a conversational interface for policymakers based on Results First clearinghouse content. During early phases, we conducted structured consultations with 13 current or former government officials and staff from professional policy associations to identify key use cases, query types, and decision-support needs. The AI assistant was built using OpenAI’s GPT-based architecture, fine-tuned on structured Results First data such as program summaries, cost–benefit metrics, and meta-analytic ratings, to ensure factual grounding and relevance. Internal and external testers provided structured feedback on early versions, guiding refinements in usability, evidence presentation, and alignment with policy workflows. A soft public launch enabled further user engagement, onboarding, and feedback collection. Full documentation of these stages and associated design principles is provided in Appendix A, including a visual schematic of the iterative process. The five AIRE Protocol stages are defined as follows:

- Needs Assessment & Contextual Inquiry: Initial interviews and surveys with policymakers identify informational gaps and clarify how AI tools can support real-world decision-making. This stage ensures that development is grounded in a clear understanding of user priorities, contexts, and challenges.
- Co-Design & Ideation: Participatory engagement with policymakers, analysts, and technical developers facilitates brainstorming of desired features and functionalities. Co-design activities focus on translating user needs into actionable design elements, with emphasis on usability, clarity, and accessibility.
- Rapid Prototyping & Development: Agile development cycles produce iterative prototypes of the AI tool. Each version incorporates direct user feedback to refine interface design, search capabilities, and evidence presentation. The assistant was developed to enable conversational querying of the Results First Clearinghouse for program-specific information.
- Living Lab Testing: Functional prototypes are deployed in real-world legislative environments. Policymakers interact with the tool during active decision-making processes, providing structured qualitative and quantitative feedback on usability, relevance, and trust.
- Deployment & Continuous Improvement: Informed by testing results, the AI tool is refined for broader deployment. Ongoing feedback mechanisms and periodic updates support continuous alignment with evolving legislative priorities and the latest evidence.

### Results First Clearinghouse Case Study: Application of the AIRE Protocol

The Results First Clearinghouse, developed by The Pew Charitable Trusts, aggregates program evaluation results across multiple domains, providing policymakers with evidence on the effectiveness various interventions. While the clearinghouse offers a valuable resource, its complexity to anyone not familiar and the time required to navigate it can pose barriers to timely decision-making, making it an ideal context for AI-assisted solutions. The Results First Clearinghouse was selected for this case study due to its status as one of the most comprehensive evidence repositories available to state and local policymakers. It synthesizes program evaluations from nine independent clearinghouses, offering rigorous assessments of interventions in key policy areas such as criminal justice, education, behavioral health, and child welfare. The Clearinghouse uses a standardized rating system, providing users with information on program effectiveness and, when available, cost–benefit analyses. Previous work considering the utility of clearinghouses have indicated a number of limitations, including (1) complex navigation: policymakers often found it challenging to locate program-specific information quickly, especially during time-sensitive legislative sessions; (2) information overload: while comprehensive, the volume of data and varying rating criteria from different source clearinghouses created confusion; and (3) limited customization: users desired tailored information relevant to their state’s context, budget constraints, and policy priorities. These challenges present a compelling case for applying the AIRE Protocol to develop an AI-driven assistant that streamlines access to the clearinghouse, presents information in a user-friendly format, and delivers tailored recommendations. The clearinghouse’s structured and validated data make it an ideal foundation for building an AI tool that prioritizes accuracy, transparency, and relevance—core pillars of the AIRE Protocol. The development of the Results First AI Assistant followed the five stages of the AIRE Protocol, ensuring a user-centered and evidence-based approach: By leveraging the structured data of the Results First Clearinghouse, the AIRE Protocol ultimately facilitates the creation of an AI tool that not only improves information accessibility but also supports evidence-informed decision-making in a high-stakes policy area.

### Evaluation of AI Tool User Behavior

After public release of the AI tool, we collected anonymous user data on tool use (e.g., requests, responses). We first employed an inductive thematic analysis to develop the coding framework. Initially, two researchers independently reviewed samples of the AI assistant discussion logs to identify recurring topics and interaction patterns. These open-coded initial themes were then discussed and compared, merging similar ideas and eliminating redundancies. Through iterative refinement, this process produced a structured coding scheme with clear categories and subcategories representing all major themes present in the data. The final codebook comprised three overarching categories, each with multiple subthemes. This included policy and program areas that encompass the subject matter of user queries, such as specific policy domains or intervention topics (e.g., Substance Use Prevention programs and other public health or education initiatives). We also coded user Engagement Trends that capture how users interact with the assistant, including patterns of query formulation and follow-up (e.g., Query Refinement behaviors where users rephrase questions or narrow their request). Lastly, we assessed evidence-based inquiry patterns that reflect the extent to which users seek validated or high-quality information (e.g., Explicit Requests for Highly Rated Programs and other indications of searching for evidence-backed answers). All user queries and related assistant responses were systematically coded using this framework. Each interaction was labeled with one or more relevant subcategory codes under the appropriate main category. We then calculated frequency counts for each code to quantify prevalent themes, and we qualitatively examined coded segments to extract illustrative examples and insights. This combined quantitative–qualitative approach enabled a clear interpretation of user needs and behaviors reflected in the logs.

## Results

The results of this study are presented in two parts. First, we report findings from a national survey of 45 US state legislators, examining their use of AI in policymaking, perceived benefits and risks, and factors influencing adoption. These findings provide insights into the current landscape of AI use among policymakers and highlight the complexity of attitudes toward emerging technologies in legislative contexts. Second, we present the application of the AIRE Protocol in developing the Results First AI Assistant, an AI-driven tool designed to enhance policymakers’ access to evidence-based information from the Results First Clearinghouse. This section details how the protocol guided the tool’s development, from initial needs assessment through prototyping, testing, and deployment. Together, these results illustrate both the existing challenges legislators face in utilizing AI and the potential for specialized AI tools to improve evidence-informed decision-making in prevention policy.

### Sample Demographics

The final sample included currently serving state legislators from across the United States. Of the participants, 59% identified as male. In terms of political affiliation, 43% identified as Democrats, 37% as Republicans, and 20% as Independents. Approximately one-third (34%) served in the state senate, with the remainder serving in state houses of representatives. Legislators had, on average, 4.74 years of experience in office (SD = 3.77), with a range from first-year legislators to those with up to 13 years of service. The average age of participants was 51.11 years (SD = 12.40), with ages ranging from 34 to 68.

### Findings from the Legislator AI Adoption Survey

#### AI Adoption Trends

**Fig. 1:**
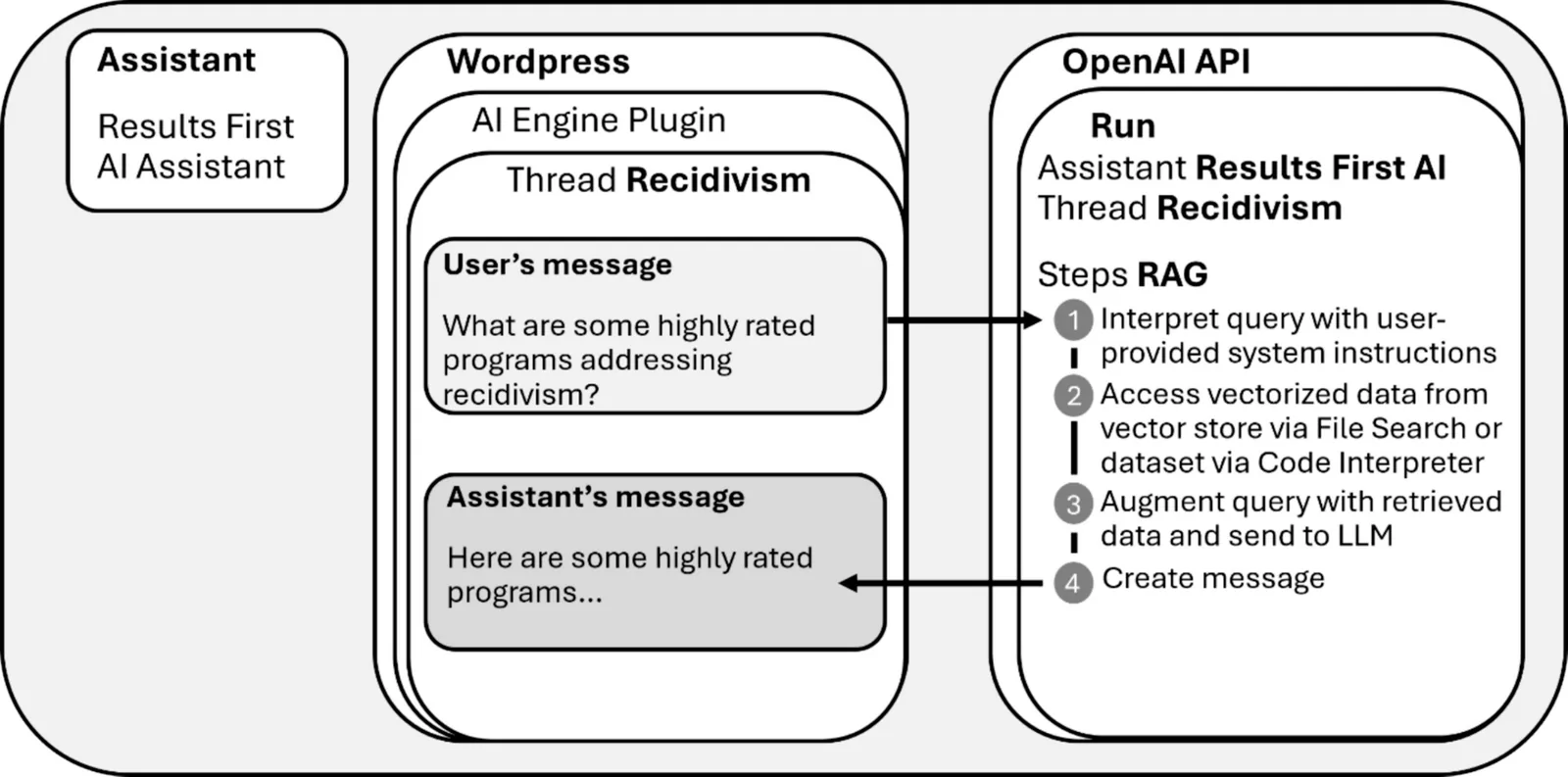
Results First Clearinghouse AI assistant architecture

Among the 45 state legislators interviewed, the survey revealed varying levels of AI adoption. When asked about their use of AI tools in their legislative work, 8 legislators (21%) openly acknowledged using AI in some capacity, 21 legislators (47%) reported not using AI, and 16 legislators (35%) preferred not to disclose their AI use (Fig. 1). These findings indicate a notable proportion of legislators who either engage with AI or are hesitant to discuss its use, suggesting complex attitudes surrounding technology adoption in policymaking. The high percentage (35%) of legislators who preferred not to discuss their AI use is noteworthy. This hesitancy may reflect uncertainty about the implications of AI use in public service, potential concerns about public perception, or institutional guidelines about technology use. These results underscore both the growing relevance of AI in legislative environments and the need for clear guidance and trusted tools to support responsible adoption.

### AIRE Protocol: Developing an AI Assistant for the Results First Clearinghouse

#### Stage 1: Needs Assessment Outcomes

The initial phase of the AIRE Protocol focused on understanding the information needs and decision-making contexts of policymakers. Engagement with 13 policymakers—including state legislators and policy advisors—revealed key challenges in accessing timely, evidence-based information on prevention programs. Participants noted that while clearinghouses like the Results First Clearinghouse offer valuable resources, navigating these databases can be time-consuming, especially under the pressures of the legislative calendar. Legislators expressed a need for tools that could quickly deliver program-specific information, implementation details, and evidence ratings. They emphasized the importance of a user-friendly interface that would allow non-technical users to efficiently obtain actionable data. Concerns about information overload and the credibility of sources further underscored the importance of ensuring that AI-generated outputs are transparent and grounded in validated evidence.

#### Stage 2: Co-Design Insights

Building on the needs assessment, the co-design phase involved direct collaboration with policymakers and representatives from professional organizations such as the National Conference of State Legislatures (NCSL) and the Council of State Governments (CSG). These sessions focused on translating user needs into practical design features for the AI assistant. Policymakers prioritized features that would allow for (a) quick searches for specific program information, (b) summaries of evidence quality and program effectiveness, and (c) tailored presentation of relevant evidence and program options aligned with legislative priorities. Such features were seen as supporting, not replacing, legislators’ judgment by improving access to relevant evidence under time constraints. Participants stressed the importance of transparency regarding how the AI tool sources and processes information. The need for a tool that could be easily integrated into existing legislative workflows—without requiring extensive technical training—was a consistent theme. Feedback from these sessions informed the development of the initial prototype.

#### Stage 3: Rapid Prototyping Process

The prototype of the AI assistant was developed using an agile, iterative approach. The tool was designed to interface directly with the Results First Clearinghouse database, allowing users to input queries related to programs and receive concise, evidence-based summaries. To validate the prototype’s usability and relevance, it was shared with seven government officials across state and federal levels, including representatives from both executive and legislative branches. User testing sessions involved guided walkthroughs of the tool’s features, followed by structured interviews to capture feedback on functionality, clarity, and overall user experience. User feedback highlighted the importance of simplifying technical jargon, improving response times, and enhancing the visual presentation of data. Legislators appreciated the transparency features but requested clearer explanations of how the AI model weighted different sources of evidence. This feedback informed subsequent iterations of the tool, with each version undergoing internal testing to refine usability and improve information delivery.

#### Stage 4: Living Lab Testing Results

Following refinements to the prototype, the AI assistant was soft-launched to a broader audience of policymakers and legislative staff for real-world testing. This living lab approach allowed users to engage with the tool during actual decision-making scenarios, providing practical insights into its effectiveness and limitations. Feedback from the living lab phase was overwhelmingly positive, with users citing significant improvements in their ability to quickly access relevant program data. Policymakers reported that the tool saved time compared to traditional research methods and facilitated more informed discussions during legislative sessions. Areas identified for further improvement included expanding the database to include additional intervention domains and enhancing the tool’s capacity to handle complex, multi-faceted queries. User feedback from this phase was integrated into the final iteration of the AI assistant.

#### Stage 5: Pilot Deployment and Public Launch

In October 2024, the finalized version of the AI assistant was officially launched to the public. The launch followed an additional round of user testing that addressed final usability issues and ensured the tool’s readiness for broader deployment. While policymakers, legislative staff, and public users have long had access to the Results First Clearinghouse through traditional search and download interfaces, they now have access to an AI-driven interface that delivers validated, evidence-based information on a wide range of prevention programs. The AI assistant now provides an alternative, conversational mode of accessing evidence. Early adoption metrics indicate steady usage across multiple states, with initial feedback suggesting that the tool is being utilized in budget discussions, program evaluations, and policy development processes. Users have commended the tool’s ease of use, quick response times, and clear presentation of complex data. The AI assistant is now integrated into the broader ecosystem of policy resources provided by professional organizations such as NCSL and CSG, further enhancing its accessibility. Plans are underway to conduct follow-up evaluations to assess long-term impacts on policy decision-making and to explore opportunities for expanding the tool’s capabilities. The development and successful launch of the AI assistant demonstrate the potential of the AIRE Protocol to deliver practical, user-centered AI solutions that bridge the gap between scientific evidence and policymaking.

### Results First Clearinghouse AI Tool User Behavior Themes

We identify key patterns and themes of how the AI tool was used in organizing around the policy and program areas, trends in user engagement, and the inquiry patterns around evidence seeking and use in Table 1.

**Table 1:** Results First Clearinghouse AI assistant

Category	Subcategory	% of Total queries	Sample user query
**Policy and Program Areas**	**Substance Use Prevention**	35%	“What are some evidence-based substance abuse prevention programs?”
	**Mental Health**	10%	“Can you recommend any programs that treat anxiety in adults ages 21–29?”
	**Criminal Justice & Public Safety**	20%	“What are some highly rated programs that help to address recidivism in youth?”
	**Education & Youth Development**	10%	“Can you recommend programs related to the use of LLMs in university learning?”
	**Child & Family Welfare**	10%	“Are there any programs that support youth who have aged out of the foster care system?”
	**Healthcare & Public Health**	15%	“What are proven strategies to increase public health insurance enrollment?”
**User Engagement Trends**	**Query Refinement**	10%	“more specific about generative AI ethical use in higher education governance”
	**Repeated Searches**	15%	“Give me examples of highly rated programs related to human trafficking in southeast United States”
	**Disengagement Points**	5%	“What transportation programs are most highly rated?”
	**Deep Engagement**	15%	“Tell me more about #3 – what are the concrete steps my nonprofit can take to effectively enroll people in a state insurance program?”
**Evidence-Based Inquiry Patterns**	**Explicit Request for Highly Rated Programs**	20%	“I’m looking for highly rated programs that deal with recidivism.”
	**Scientific Rigor** **(RCTs, study quality)**	5%	“How many RCTs were available for medication-assisted treatment? What’s the quality of the evidence? What’s the sample size for individual studies?”
	**Broad Landscape Queries (Total Programs)**	10%	“How many programs are there that address AI and data privacy?”

#### Policy and Program Areas

The user queries spanned a range of policy and program domains, with certain topics dominating. Substance Use Prevention was the most frequent focus, accounting for the largest share of queries (e.g., requests for drug misuse prevention programs). Mental Health was the next most common area, including queries about anxiety treatment and other behavioral health interventions. Education-related inquiries (often intersecting with technology ethics in education) also featured prominently—for example, users asked about programs addressing AI governance in higher education. Other policy areas appeared less frequently, including Crime and Justice (e.g., youth recidivism, human trafficking programs) and Public Health/Economic issues (e.g., strategies to increase health insurance enrollment, community economic development). Each major category is illustrated by typical queries as noted above, reflecting the distribution of interests across these topics.

#### User Engagement Trends

Analysis of the interaction logs revealed distinct user engagement patterns. Query Refinement was common: many users rephrased or narrowed their searches when initial results were too broad or not perfectly aligned. For instance, one user iteratively specified their query about AI and data privacy—first generally, then focusing on education, then on higher education. Such stepwise refinement occurred in over half of the sessions. Repeated Searches were also observed, with some users conducting multiple related queries in a single session (often exploring different facets of a topic). In about one-third of cases, users showed Deep Engagement, asking follow-up questions to dig into details of the results. For example, after receiving a list of programs, users would ask for more information on specific items (“tell me more about #3 …”) or request outputs (e.g., asking for a downloadable list). Conversely, a subset of sessions (approximately one-fifth) were one-off interactions where the user posed a single question and did not continue, indicating a possible disengagement after getting an initial answer.

#### Evidence-Based Inquiry Patterns

Users demonstrated a strong preference for evidence-based information. The majority of queries explicitly requested high-quality, validated programs—for example, asking for “highly rated” interventions in a given area or specifically using the term “evidence-based programs” in their request. Approximately two-thirds of the queries included such evidence-oriented language, highlighting users’ intent to find programs backed by proven results or scientific rigor. Moreover, several users engaged in broad landscape inquiries to gauge the evidence base, asking how many programs exist for a particular issue or what the overall evidence quality is. For instance, users queried the number of randomized controlled trials (RCTs) available for certain treatments and the quality of that evidence. These patterns underscore that users were not just seeking any information but specifically aiming to identify interventions with strong evidence of effectiveness, as well as obtaining a broad overview of the evidence landscape for informed decision-making.

## Discussion

This study provides new insights into the adoption of artificial intelligence by US state legislators and demonstrates the practical application of the AIRE Protocol in developing an evidence-based AI assistant. The survey results indicate that while a minority of legislators (21%) openly use AI tools, a substantial proportion (35%) preferred not to disclose their use, suggesting complex attitudes toward AI adoption in policymaking. The reluctance to discuss AI use may stem from concerns about public perception, institutional policies, or uncertainty regarding the technology’s implications. Notably, the sizable group of legislators hesitant to acknowledge AI use underscores the need for transparent, trusted tools that align with legislative norms and ethical standards. The findings also reveal that efficiency is a key motivator for those who use AI, with legislators citing time savings and improved information access as primary benefits. However, concerns about data privacy, algorithmic transparency, and potential bias were also expressed among AI users. Overall, our data do not suggest that legislators seek to place policy decision responsibility on AI systems. Rather, across our data collection, participants described AI as a supplemental research aid whose outputs require human judgment and accountability. These findings align with broader literature on technology adoption in public policy, which highlights the tension between leveraging innovative tools and maintaining public trust and accountability.

### Lessons Learned

The successful application of the AIRE Protocol in developing the Results First AI Assistant illustrates how user-centered, evidence-based AI tools can address policymakers’ needs while mitigating concerns associated with generalized commercial AI solutions. By engaging policymakers throughout the development process, the AIRE-guided approach ensured that the tool was practical, transparent, and aligned with legislative workflows. Early adoption and positive user feedback suggest that such specialized tools can improve the integration of validated scientific evidence into the policymaking process.

Thematic analysis revealed that users consistently gravitated toward evidence-based programs and interventions with high evidence ratings. This preference for validated, high-quality sources suggests that policymakers using AI tools expect recommendations grounded in credible research. Additionally, user engagement trends—such as frequent query refinements and repeated searches—indicate that users often had to iterate to find relevant information. This pattern highlights an opportunity for AI tools to improve responsiveness and personalization so that users can obtain the needed evidence more efficiently without multiple attempts.

These findings point to several design improvements for AI systems supporting policymaking. First, AI tools should better accommodate iterative search behaviors by allowing users to refine queries seamlessly (e.g., through interactive filters or follow-up prompts). Enhanced filtering mechanisms (by topic, target population, or evidence strength) and clear explanations of evidence ratings would help users quickly narrow down credible options. Ensuring transparency in how results are generated—for instance, indicating why a program is recommended—can further build user trust. Integrating feedback loops is also key: AI systems can learn from common query refinements and continuously optimize search results to align with user needs over time.

### Implications for Prevention Policy

The integration of AI into policymaking has significant implications for the development and implementation of prevention policies. Prevention science relies on the timely use of high-quality evidence to inform decisions about which interventions to fund, scale, or discontinue. However, policymakers often face barriers in accessing and interpreting this evidence. The Results First AI Assistant, developed using the AIRE Protocol, addresses this challenge by providing legislators with direct, user-friendly access to validated program evaluations and cost–benefit analyses. By streamlining the process of obtaining evidence-based information, the AI assistant has the potential to enhance the selection of effective interventions, ultimately improving outcomes for children, families, and communities. For instance, legislators making budgetary decisions can now more readily compare the effectiveness of prevention programs, leading to more informed allocations of public resources. This capacity is particularly important in contexts where legislative sessions are short, and decision-making timelines are compressed. The development of the AI assistant also highlights the importance of partnerships between researchers, technology developers, and policymakers. Such collaborations are essential for creating tools that not only meet technical standards but are also practical and responsive to the realities of policymaking environments.

### Ethical and Practical Considerations

The use of AI in policymaking raises several ethical and practical considerations that must be addressed to ensure responsible deployment. A primary concern is the potential for algorithmic bias, which can occur if the data used to train AI models reflect existing inequities. To mitigate this risk, the AIRE Protocol emphasizes the use of validated, high-quality data sources, such as the Results First Clearinghouse, and includes iterative testing with diverse user groups to identify and address potential biases. Transparency is another critical consideration. Policymakers must be able to understand how AI-generated recommendations are produced, particularly when these tools influence decisions with significant public health and economic implications. The AI assistant developed in this study includes features designed to enhance transparency, such as explanations of data sources, the methodology used to evaluate programs, and the rationale behind confidence scores assigned to recommendations. Also, the AI assistant was designed to return only documents and data from its external store, which minimizes the possibility for the bot to return incorrect, irrelevant, or hallucinated answers. Further design would include the implementation of chain-of-thought and information sourcing that is made possible by the underlying language models used for the AIRE Protocol. These features are essential for fostering trust and encouraging responsible use. Data privacy and security also emerged as important concerns during the development and testing phases. Legislators emphasized the need for robust safeguards to protect sensitive information, particularly when using AI tools to analyze proprietary or confidential data. The AI assistant was designed with strict data privacy protocols, ensuring compliance with relevant regulations and best practices in cybersecurity. Practical considerations, such as ease of use and integration into existing workflows, are equally important. Policymakers operate in high-pressure environments with limited time, making it essential that AI tools are intuitive and require minimal training. The user-centered design process embedded in the AIRE Protocol addressed this need, resulting in an interface that legislators found accessible and straightforward.

The AIRE Protocol offered a distinctive advantage over more generic AI development and implementation science frameworks by explicitly centering the end-user—in this case, policymakers—as both co-designers and validators of the AI assistant. Unlike general lifecycle models for AI or knowledge translation, AIRE integrates a policy-specific lens at each stage, ensuring that technical development is continuously aligned with real-world constraints, language use, and trust concerns faced by public officials. Notably, users reported employing the Results First AI Assistant in contexts central to the legislative process, including drafting talking points for bill sponsors, preparing for appropriations hearings, and comparing investment options during budget negotiations. This tight integration with policy workflows suggests that AI tools developed under the AIRE framework can serve as embedded, just-in-time supports within core decision-making activities, thereby enhancing the uptake and utility of evidence-based prevention strategies. Future refinement of the Results First AI Assistant will be conducted in partnership with subject matter experts in education, criminal justice, and public health to ensure technical accuracy and contextual relevance. The AIRE Protocol explicitly includes co-design activities with both policy stakeholders and domain experts to guide iterative development.

### Limitations

While this study provides valuable insights into AI adoption among state legislators and demonstrates the utility of the AIRE Protocol, several limitations warrant consideration. First, the survey sample size of 45 legislators, while diverse, limits the generalizability of the findings. Although participants represented various states, political affiliations, and levels of experience, the results may not fully capture the breadth of perspectives across all US state legislatures. Future research should aim to include larger and more representative samples to validate and extend these findings. Second, the reliance on self-reported data introduces the potential for response bias. Legislators may have underreported or overreported their use of AI due to concerns about public perception, institutional guidelines, or a lack of familiarity with the technology. The notable proportion of respondents who preferred not to disclose their AI use underscores this challenge. Third, while the development and deployment of the Results First AI Assistant were guided by user feedback, the study focused primarily on the tool’s initial rollout and short-term adoption. Long-term evaluations are needed to assess sustained use, the tool’s impact on policy decisions, and any unintended consequences that may arise from its integration into legislative processes. Finally, the AI assistant was designed to interface with a specific evidence base (the Results First Clearinghouse). While this focus ensured the use of validated data, it may limit the tool’s applicability to other policy domains not covered by the clearinghouse. Future iterations should explore opportunities to expand the tool’s data sources while maintaining rigorous standards for evidence validation.

### Future Directions

Building on the promising early adoption of the Results First AI Assistant, several avenues for future work are recommended. First, expanding the tool’s capabilities to include additional data sources and intervention domains could broaden its utility for policymakers. For example, integrating databases related to education, criminal justice, and public health could provide a more comprehensive resource for legislators addressing complex, cross-sector policy issues. Second, long-term evaluations are needed to assess the AI assistant’s impact on policy decision-making processes and outcomes. Future studies should examine whether access to the tool leads to increased adoption of evidence-based interventions, improved allocation of resources, and measurable benefits for target populations. Such evaluations would provide critical evidence on the tool’s effectiveness and inform ongoing refinement. Third, further exploration of the ethical implications of AI use in policymaking is warranted. While the AIRE Protocol addresses key concerns related to transparency and bias, continued engagement with ethicists, community stakeholders, and policymakers is essential to navigate emerging challenges. Issues such as algorithmic accountability, equity in access to AI tools, and the potential for overreliance on automated recommendations should remain central to future development efforts. Fourth, efforts to scale the tool beyond state legislatures to include federal policymakers, local governments, and international policy bodies could enhance its reach and impact. Collaborations with organizations such as the National Conference of State Legislatures and the Council of State Governments have demonstrated the value of partnerships in facilitating tool dissemination and adoption. Finally, as AI technology evolves, continuous improvement processes must be maintained to ensure that the AI assistant remains aligned with best practices in both prevention science and AI development. Regular updates to the tool’s evidence base, user interface, and algorithmic components will be essential to sustain its relevance and effectiveness in dynamic policymaking environments.

## Data Availability

Data available upon request to the corresponding author.

## Appendix

### A1. Overview of AIRE Protocol

The AI for Informed and Responsible Evidence-use (AIRE) Protocol is a structured, human-in-the-loop framework designed to guide the development of artificial intelligence tools that translate validated scientific knowledge for use in real-world decision-making. Developed in response to growing demand for AI systems that are transparent, policy-relevant, and evidence-grounded, the AIRE Protocol provides a repeatable, stage-based roadmap for creating AI tools that reflect the priorities, workflows, and ethical standards of end users—particularly within the public sector. In the development of the Results First AI Assistant, the AIRE Protocol was applied to ensure that the system not only integrated credible evidence from the Results First Clearinghouse, but also aligned with the informational needs and contextual realities of policymakers. The protocol includes five stages: Needs Assessment & Contextual Inquiry, Co-Design & Ideation, Rapid Prototyping & Development, Living Lab Testing, and Deployment & Continuous Improvement. These stages are detailed below and visually summarized in Figure A1.

### A2. Protocol Goals and Design Principles

The AIRE Protocol was created to ensure that artificial intelligence tools supporting evidence-based decision-making are purpose-built for real-world use by public leaders. It aims to close the gap between scientific knowledge and policymaking by integrating end-user insights throughout the AI development lifecycle. Unlike generic AI development workflows, AIRE emphasizes grounding in established evidence bases, collaboration with decision-makers, and ongoing adaptation to evolving policy contexts.

The protocol is anchored in five core design principles:

- Scientific Integrity—AI outputs must reflect evidence from rigorously evaluated sources, such as clearinghouses or peer-reviewed syntheses.
- Policy Relevance—Development is guided by policymaker priorities and real-world decision scenarios to maximize applied value.
- Usability and Trust—Tools are designed to be intuitive, responsive, and transparent to promote user trust and sustained engagement.
- Iterative Co-Development—user feedback is continuously integrated at each stage, improving tool quality and relevance over time.
- Sustainable Adaptation—Deployment includes mechanisms for continuous improvement based on user behavior, policy changes, and updates to the evidence base.

These principles shaped each stage of the Results First AI Assistant’s development and serve as the foundation for its technical and user-facing design features.

### A2.1 Needs Assessment & Contextual Inquiry

The development of the Results First AI Assistant began with structured interviews and surveys with 13 current or former government officials and policy association staff. These participants represented both legislative and executive branches across diverse U.S. states and included Democratic, Republican, and Independent affiliations. Conversations focused on identifying pressing informational needs during key phases of the policymaking process, including bill drafting, budget negotiation, and program oversight. Participants shared perspectives on how AI could be integrated into existing workflows and identified limitations of current evidence tools. Feedback revealed a need for intuitive, natural language interfaces capable of quickly translating validated evidence into policy-relevant insights. Usability concerns, especially around trust in AI-generated responses, were prominent. This stage clarified the types of queries legislators might pose to an AI assistant and surfaced the importance of grounding all outputs in recognizable and authoritative sources, such as the Results First Clearinghouse. Findings directly shaped the Assistant’s initial design scope.

### A2.2 Co-Design & Ideation

Building on initial interviews, we convened a series of co-design workshops with a multidisciplinary team of prevention scientists, legislative staff, policy researchers, and AI developers. Participants collaboratively mapped user journeys and articulated specific decision-making pain points, such as difficulty comparing programs or locating cost–benefit estimates. These workshops helped translate user needs into concrete system requirements, including evidence summarization, side-by-side program comparisons, and citation links. Key design decisions—such as the use of plain-language responses, embedded definitions, and transparency tags—emerged from these sessions. The design team placed strong emphasis on minimizing jargon and presenting complex economic and evaluative data in formats familiar to legislative audiences. Policymakers also stressed the importance of conversational flexibility while ensuring accuracy, which led to early integration of guardrails to flag limitations in AI responses. Insights from co-design directly informed technical implementation priorities, UI/UX specifications, and the assistant’s tone and structure of interaction.

### A2.3 Rapid Prototyping & Development

The Assistant’s development proceeded through a series of agile sprints, each focused on refining functionality, accuracy, and user responsiveness. An internal quality assurance team conducted repeated cycles of prompt testing to assess model outputs under realistic legislative queries. Technically, the Assistant leveraged OpenAI’s GPT-based large language model, fine-tuned on structured Results First datasets, including program descriptions, cost–benefit tables, and evidence quality ratings. Fine-tuning ensured that outputs reflected the structure, terminology, and framing used in the clearinghouse. System prompts and few-shot examples were adjusted to prioritize conservative summarization and citation fidelity. The Assistant was designed as a web-based, open-access tool, compatible with screen readers and mobile devices. Open-source documentation and modular code were maintained to facilitate future expansion. Development iterations prioritized functionality that directly addressed use cases identified during the earlier stages, including answering “Which program is best for…?” and “What’s the ROI on…?” types of questions with clear, sourced responses.

### A2.4 Living Lab Testing

Functional prototypes of the AI assistant were tested in live government settings through structured engagements with seven state and federal policymakers and staff. Participants used the tool during real-world scenarios—e.g., preparing committee testimony, drafting constituent communications, and reviewing funding proposals. Structured post-use interviews captured impressions of usability, accuracy, and policy relevance, while backend telemetry tracked types of queries submitted. Feedback underscored the importance of clarifying data provenance and offering transparent citations, which led to improvements in how cost–benefit ratios and evidence quality were displayed. Users highlighted the tool’s potential for improving efficiency during legislative research, especially under time constraints. Living lab users also flagged specific policy domains (e.g., education, early childhood) where Results First content was especially valued, prompting refinement of query recognition and summary generation for these domains. These sessions provided critical insights that informed both backend prompt tuning and user-facing features prior to full launch.

### A2.5 Deployment & Continuous Improvement

Following living lab testing, the Results First AI Assistant entered a soft launch period in October 2024. The tool was deployed publicly through a dedicated web portal, accompanied by a suite of onboarding materials including explainer videos, a user guide, and training slide decks. Early users included legislative staff, state agency analysts, and national policy organizations. Continuous feedback mechanisms were established through an embedded comment feature, optional user surveys, and follow-up interviews. An internal team monitored usage patterns and response fidelity, initiating regular prompt updates and model recalibrations where needed. Planned updates include expanding coverage beyond Results First content, integrating additional clearinghouse datasets, and offering customized dashboards for recurring users. The tool remains freely accessible and is actively promoted through national networks, such as the National Conference of State Legislatures. Ongoing refinement follows the AIRE Protocol’s emphasis on real-world testing, transparency, and co-ownership by intended end-users.

### A3. Design Principles & Ethics Considerations

The AIRE Protocol was developed to guide the creation of AI tools that are transparent, factual, safe, and usable in real-world policy contexts. Design principles were operationalized throughout the Results First AI Assistant’s development, emphasizing interpretability of responses, clear citation of data sources, and strict alignment with validated evidence. Human oversight was embedded at every stage: stakeholder consultations informed early scoping, co-design ensured user-centered feature development, and all outputs underwent manual review during prototyping and deployment. Internal testers flagged inaccuracies or hallucinations, triggering prompt and model adjustments. While this study did not include direct probing of policymakers’ ethical concerns about AI—an omission noted in our reviewer feedback—our future work will incorporate structured assessments of ethical risks, including trust, bias, and fairness, using established AI ethics frameworks. These considerations will inform subsequent development cycles and expanded applications of the AIRE Protocol in Table 2.

The Results First Clearinghouse is accessible online at https://evidence2impact.psu.edu/results-first-resources/clearing-house-database/

**Table 2:** Survey respondents’ demographic characteristics

Characteristic	Category	Descriptive
**Gender**	Male	59%
**Party affiliation**	Democrat	43%
	Independent	20%
	Republican	37%
**Legislative chamber**	Senate	34%
**Years in office**	Mean ± SD	4.74 ± 3.77
	Range	1–13
**Age**	Mean ± SD	51.11 ± 12.40
	Range	34–68

## Declarations

### Clinical Trial Registration

NCT06856148 and initial release date 2/19/2025.
